# Low-Grade Central Osteosarcoma of the Rib: A Case Report and Brief Review of the Literature

**DOI:** 10.1155/2013/798435

**Published:** 2013-05-14

**Authors:** Mana Moghadamfalahi, Houda Alatassi

**Affiliations:** Department of Pathology and Laboratory Medicine, 503 South Jackson Street, Louisville, KY 40202, USA

## Abstract

Low-grade central osteosarcoma is a rare variant of osteosarcoma which comprises less than 1-2% of all osteosarcomas. Most low-grade osteosarcomas involve long bones, most commonly distal femur, and proximal tibia. Histologically this tumor is difficult to diagnose, and an unusual location makes this diagnosis even more challenging. Here we report a case of low-grade osteosarcoma presenting as a chest wall mass involving the left 6th–8th ribs. This unusual site of presentation significantly added to the diagnostic difficulties of this rare tumor with challenging histologic features. To the best of our knowledge, only six cases of low-grade central osteosarcoma of the ribs have been reported in the English literature.

## 1. Introduction

Low-grade central osteosarcoma (LGCO) is a very rare variant of osteosarcoma. The most common location is the long bones with the distal femur and proximal tibia being the most common sites [1].

Rare incidence and overlapping pathological and radiological features of this tumor make the diagnosis difficult. 

We present a rare case of LGCO of the rib in a 33-year-old male with a brief review of the literature and emphasize on diagnostic difficulties, differential diagnosis, and treatment. Six other cases of LGCO of the rib have been previously reported in the English literature [2, 3].

## 2. Case Report

A 33-year-old male presented with a gradually growing mass in his chest wall. The mass was involving the left seventh rib in approximately the mid to anterior axillary line. 

**Figure 1:**
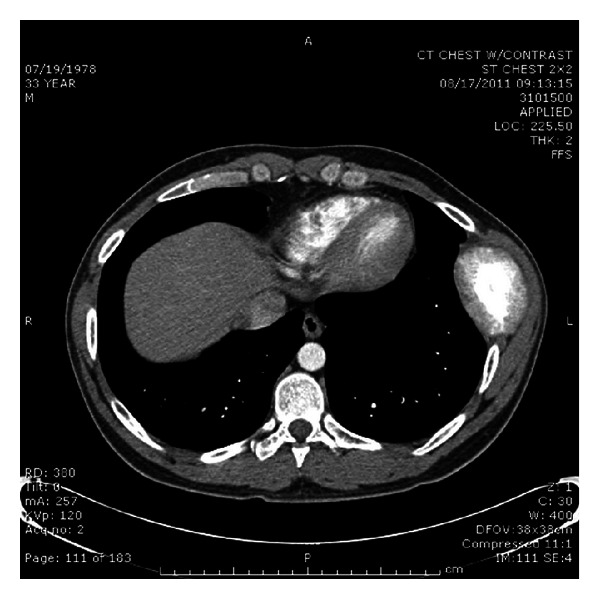
CT scan of the chest showing a large mass of the left lateral aspect of the seventh rib with bony matrix and sunburst central core.

A chest computed tomography (CT) scan revealed a large left lateral 7th rib mass with an osteoid matrix and sunburst pattern, highly suspicious for a primary osteosarcoma (Figure 1).

**Figure 2:**
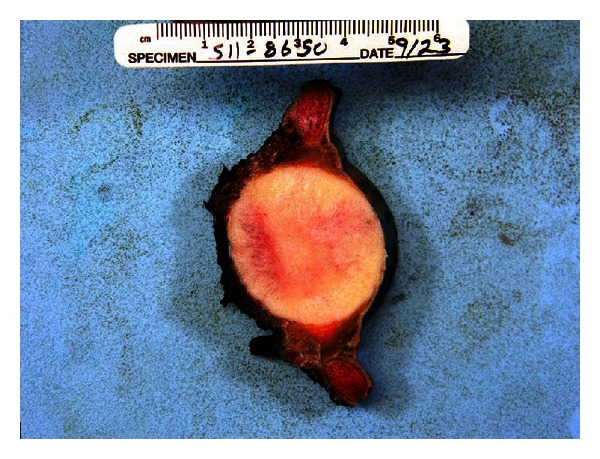
Gross examination of the mass showing a tan white hard mass involving the medullar cavity of the rib with extension to adjacent soft tissue.

An enbloc resection of the mass was performed. Gross examination revealed a 4.0 cm hard mass with a gray-white gritty cut surface, predominantly involving the medullary cavity of the seventh rib with extension to the adjacent sixth and eighth ribs (Figure 2). No necrosis or hemorrhage was identified.

**Figure 3:**
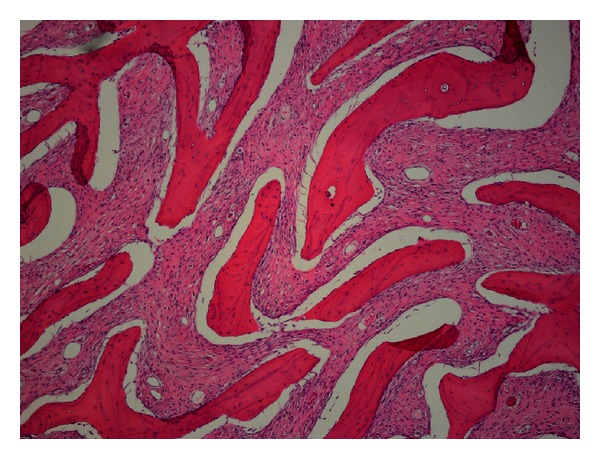
Microscopic examination of the mass revealed moderately atypical fibroblastic cells surrounding parallel trabeculae of woven bone.

Microscopic examination showed long sweeping fascicles of moderately atypical fibroblastic cells surrounding streamers of woven bone. No necrosis identified. Rare mitotic figures are noted (Figure 3). The tumor extended through the rib into the surrounding soft tissue.

## 3. Discussion

Low-grade central osteosarcoma (LGCO) is a very rare tumor and was first described in 1977 by Unni et al. [3].

Most of the patients present with a slow-growing mass with associated pain. Males and females are equally affected.

Patients with LGCO are older than those with conventional osteosarcoma with a peak incidence in the third decade of life [4].

The most common site of involvement is the intramedullary cavity of metaphysis or diametaphysis of the long bones [1]. Rare cases have been reported from occipital bone, mandible, and intracranial sphenoid bone [4].

This tumor has a significant better prognosis in comparison to conventional osteosarcoma. Treatment with wide local excision and resection essentially is curative versus marginal excision or curettage which almost always results in recurrence with possible transformation into a high-grade dedifferentiated osteosarcoma in about 15% of the cases [5, 6]. Therefore, accurate diagnosis is an essential component in survival of these patients.

Initial diagnosis of this tumor in a small biopsy can be extremely challenging, and multiple biopsies and sometimes consult with a specialist are required in order to establish a correct diagnosis before a definitive excisional surgery is performed. In a study by Andresen et al., some cases required several biopsies in order to make the diagnosis of LGCO, even in a primary referral. Based on their experience only six of the 11 patients treated primarily had a clear diagnosis of LGCO after the first biopsy [7].

Four radiological patterns have been described for this tumor by Malhas et al. [8]. including (1) lytic with varying amounts of thick and coarse trabeculation; (2) predominantly lytic with few thin, incomplete trabeculae; (3) densely sclerotic; (4) mixed lytic and sclerotic [7].

Diagnosis on imaging study might be difficult and can look benign with usually some focal aggressive features [5].

This tumor can be confused radiologically with fibrous dysplasia, desmoplastic fibroma, nonossifying fibroma, osteoblastoma, and aneurysmal bone cysts [6–10]. 

The main differential diagnoses are fibrous dysplasia and desmoplastic fibroma [11].

The main histologic pattern differentiating this tumor from fibrous dysplasia is a permeative growth pattern encasing preexisting trabecular bone. Besides extension to the adjacent soft tissue is another feature which is not associated with fibrous dysplasia [12].

Any bone formation will exclude desmoplastic fibroma [12].

LGCO has a simple karyotype with supernumerary ring chromosomes, which are composed of 12q13-15 clusters containing *MDM2* and *CDK4* and are due to telomeric deletions [12].

In two recent studies by Yoshida et al. and Dujardin et al., it is shown that LGCOs are frequently positive for *MDM2* and *CDK4* immunostains with a very high specificity and sensitivity. These markers can be used in difficult situation especially in small biopsies to exclude the other fibrous or fibro-osseous benign lesions [11, 12].

In conclusion, LGCO is a treatable malignant tumor with a high chance of cure with appropriate preoperative diagnosis and adequate surgical treatment. Newly introduced CDK4 and MDM2 immunostains can be helpful especially in the small biopsies. Sometimes multiple biopsies and open surgical biopsies are required for an accurate initial diagnosis. In the event of difficult cases with an unusual site of presentation, like our patient, consult with an expert is necessary in order to make an appropriate diagnosis.
